# Reviewed article: Rodrigues HTB, Garcia MT. Perceptions of Family Health Strategy doctors and nurses on the promotion of adequate and healthy eating practices in light of the Dietary Guidelines: qualitative study, 2022. Epidemiol Serv Saude. 2025:34;e20240456

**DOI:** 10.1590/S2237-96222025v34e20240456.a

**Published:** 2025-06-13

**Authors:** Juliana Bicalho

**Affiliations:** UFSJ

**Table:** 

Rating	Excellent	Good	Average	Below average	Poor
Interest	✓				
Quality	✓				
Originality	✓				
Overall	✓				

**Table:** 

Questionnaire	Yes	No	Not applicable
Does the manuscript contain new and significant information to justify publication?	✓		
Does the Abstract (Summary) clearly and accurately describe the content of the article?	✓		
Is the problem significant and concisely stated?	✓		
Are the methods described comprehensively?	✓		
Are the interpretations and conclusions justified by the results?	✓		
Is adequate reference made to other work in the field?	✓		

## Manuscript Structure

Length of article is: Adequate

Number of tables is: Adequate

Number of figures is: Adequate

Please state any conflict(s) of interest that you have in relation to the review of this paper (state “none” if this is not applicable): None

## Comments

Parabéns pela relevante pesquisa sobre Alimentação Adequada e Saudável na percepção dos médicos e enfermeiros da APS. A leitura do trabalho é agradável e fluida.

Solicito atenção para revisão de alguns pontos:

Adeque, na Introdução, o termo “anos de vida ajustados por incapacidade”, pois está incompleto e o correto seria “anos de vida perdidos ajustados por incapacidade”.

Ainda na Introdução, é necessário abordar a Política Nacional de Alimentação e Nutrição de 2013, assim como é essencial contextualizar a publicação do Guia Alimentar para a População Brasileira de 2005.

